# Revealing the critical role of *in-situ* plant and microbe community structure in remediation of typical high-arsenic soil through molecular analysis

**DOI:** 10.3389/fpls.2025.1608933

**Published:** 2025-10-10

**Authors:** Min Zhou, Feng Shi, Xinru Li, Hailei Su, Yuan Wei, Fanfan Wang

**Affiliations:** ^1^ State Key Laboratory of Environmental Criteria and Risk Assessment, Chinese Research Academy of Environmental Sciences, Beijing, China; ^2^ National Center for Science & Technology Evaluation, Beijing, China; ^3^ College of Environmental Science and Engineering, Tongji University, Shanghai, China

**Keywords:** soil remediation, dominant native plant, dominant microbes, arsenic, 18S rDNA

## Abstract

*In situ* plant–fungal combined remediation technology for arsenic (As)-contaminated soil has emerged as a dominant technology in soil pollution remediation both domestically and internationally. However, the lack of systematic studies on *in situ* plants and rhizosphere fungal diversity in As-contaminated soils, particularly in heavily polluted area, limits the application of the plant–fungal combined remediation technology. In this study, we surveyed and identified the distribution of dominant native plant in highly arsenic-contaminated area, and then we used 18S rDNA technology to analyze the diversity of rhizosphere fungi and related factors from the area. The results revealed that *Pteris vittata* (L.) of Pteridaceae and *Imperata cylindrica* (L.) Beauv. of Poaceae are the dominant native plants in highly arsenic-contaminated area. The concentrations of As in the rhizosphere soils of the dominant plants in the area exceeded the As soil limits set by the European Union and the World Health Organization. A large quantity of As resulted in the dominance of fungi from the phyla *Glomeromycota*, *Ascomycota*, and *Basidiomycota* in the contaminated area soils, while relative abundance of fungi is varied in different sites. Additionally, soil acidity and alkalinity (pH), available phosphorus (AP), and As had the most notable effects on fungal diversity in Shihuangsi village and Linkuang village, whereas the low soil organic carbon (SOC) content in Heshan village was the primary limiting environmental factor for fungal diversity. The results of this study provide a theoretical foundation and technical guidance for the development of novel plant–fungal combined remediation technologies aimed to the control of As pollution in plant and soil.

## 1 Introduction

Arsenic (As) is a highly toxic metalloid element, which can enter the human body through diet, skin contact, and breathing, leading to cardiovascular disease, neurotoxicity, and skin cancer (Fayiga and Saha, 2016; Al-Makishah et al., 2020). Therefore, As is classified as a Group 1 carcinogen by the International Agency for Research on Cancer (IARC) and the World Health Organization (WHO) (Fayiga and Saha, 2016). Arsenic is widely distributed in the natural environment, with soil being the primary medium for its presence as a result of ore mining, smelting, and the use of As-containing products such as pesticides and animal feed additives (Kayode et al., 2021; Feng et al., 2024). For example, As content in the agricultural soils of the Badhshir Plain in southeastern Iran was as high as 50.26 mg/kg (Khajehpour et al., 2022), and that in the agricultural topsoil in China was as high as 3.71 × 10^6^ t (Gong et al., 2020). Soil As contamination is even more severe in mining areas. The As content in the soil of the abandoned Shimen realgar mine in China was 86.32–43,997.76 mg/kg (Chen et al., 2020). Moreover, As present in the soil can enter the food chain through plant uptake and ultimately pose a threat to human health. Therefore, addressing soil As contamination in soil has always been a major focus. Currently, traditional physical and chemical remediation techniques are being increasingly substituted with *in situ* phytoremediation, primarily for their cost-effectiveness and reduced risk of secondary pollution. For example, native dominant hyperaccumulator plants such as *Pteris vittata*, *Pityrogramma calomelanos*, and *Vetiveria zizanoides* are used to accumulate or immobilise As in their roots (Anh et al., 2018; Eze and Harvey, 2018), thus achieving ecological remediation of As-contaminated soils. However, for highly As-contaminated soils, the high concentration of As limits the growth of dominant plants, reducing phytoremediation efficiency (Wu et al., 2024; Yan et al., 2024). Therefore, it is imperative to prioritise the development of innovative and efficient technologies for the remediation of As-contaminated soils, particularly those with elevated levels of the contaminant.

In recent years, several studies have indicated that certain microorganisms, especially fungi such as arbuscular mycorrhizal fungi (AMF), can interact with plants to enhance their survival in heavy metals-contaminated soils, thereby improving the ecological remediation efficiency of soils with high concentrations of heavy metals (Gupta et al., 2021; Salari et al., 2024). For example, Cr(III) can be precipitated by the negatively charged phosphate groups (or analogues) present on the surface of AMF fungi, others can combined with histidine analogues or carboxylic acid ligands and be adsorbed onto the fungal cell wall (Wu et al., 2016). *Rhizophagus irregularis* reduced the uptake and translocation of Bi in the plant organs by enhancing the exudation of polyphenols and organic acids into the rhizospheric soil (Mohammed et al., 2023). The symbiotic network by AMF and root can effectively immobilize and capture As through chitin on the cell wall, thereby reducing the absorption of As by plants and improving the tolerance of plants to As (Khalid et al., 2021). *Claroideoglomus etunicatum* promotes the transformation of As from crystalline forms to an iron - aluminium oxides that is readily available for plant utilization in the rhizosphere soil of *Pteris vittata* (L.) by regulating plant rhizosphere exudates such as organic acids, thus activating As in the rhizosphere soil and enhancing its uptake by plants. This also changes the valence state of As in the soil, improving plant survival and increasing plant efficiency in remediating As contamination (Pan et al., 2024). As-resistant fungi such as *Fimetariella rabenhorstii* and *Hormonema viticola* can enhance plant resistance to As by upregulating genes associated with metallothioneins, superoxide dismutases, ascorbate peroxidases, and phytochelatins, thereby promoting plant growth in As-contaminated areas (Soto et al., 2019). The As-resistant *Trichoderma* sp. promotes the conversion of As from inorganic to organic forms in plants, reducing As toxicity and increasing plant tolerance to As stress (Tripathi et al., 2017). AMF participates in the process of reducing As(III) to As(V). As(III) can forms complexes with thiol compounds, ultimately being stored in plant vacuoles or excreted into the environment, thereby reducing arsenic toxicity to the plants (Zhang et al., 2015). Cantamessa et al. (2020) carried out research in a large outdoor area (an abandoned As mining site in northern Italy) to investigate the impact of AMF on the growth of *P. vittata*, and found that AMF can increase the biomass of plant. In conclusion, the formation of the aforementioned microbial-plant co-remediation system primarily involves AMF enhancing plant tolerance to heavy metals through both direct and indirect effects. Regarding direct effects, AMF forms symbiotic associations (hyphae) with plants, increasing the specific surface area of root. This promotes the uptake of mineral elements such as nitrogen (N) and phosphorus (P), thereby benefiting plant growth (Kuang et al., 2025). Simultaneously, thiol groups on their extraradical hyphae and polyphosphate within the hyphae can directly immobilize and chelate heavy metals, reducing their toxicity to plants (Chen et al., 2018; García-Sánchez et al., 2021). Regarding indirect effects, AMF can regulate rhizospheric soil environment by altering plant root exudates, such as pH, redox potential and organic matter composition, thereby affecting speciation and bioavailability of heavy metal in rhizosphere soil (Dhalaria et al., 2020). In addition, AMF can also enhance plant resistance to heavy metals by regulating changes in root morphology, increasing antioxidant enzyme activity, and modulating the expression of genes encoding heavy metal transporters (Ferrol et al., 2016; Riaz et al., 2021).

Therefore, the combination of dominant fungi and plants in soil presents a promising approach for remediating high levels of As contamination. Thus, most research on the combined use of microbes (such as AMF) and plants to remediate As-contaminated soil have been conducted under controlled greenhouse conditions (Akhtar et al., 2024; Asif et al., 2025). The application of locally advantageous As-accumulating plants and *in situ* microorganisms in practical environments has become a key constraint on the efficient remediation of As-contaminated soil. However, there is limited research on the dominant microorganisms in As-contaminated soils, and the diversity of rhizosphere fungi in these areas remain unclear. This lack of information presents challenges in identifying dominant microorganisms and hinders the development and application of fungal-plant cooperative remediation techniques for highly As-contaminated areas. Therefore, identifying the structure and composition of native dominant As-accumulating plants and *in-situ* microbial communities in typical As-contaminated soil is a necessary prerequisite for the application of the above-mentioned combined remediation methods in actual soil environments.

Based on the above background and current situation, we conducted a study on the fungal diversity in a highly As-contaminated area—the Shimenshan Realgar mining area, which is located in Shimen County, Hunan Province, and is the largest realgar mining area in Asia. The area has a mining heritage that spans over 1500 years, but mining activities ceased in 2011 (Yang et al., 2013). Due to the previous intensive mining and smelting activities, the soil in this area was severely contaminated, with the soil As content ranging from 86.32 to 43997.76 mg/kg (Chen et al., 2020). The soil As content in the surrounding Heshan Village also exhibited elevated levels, reaching as high as 5240.8 mg/kg (Tang et al., 2016). In the present study, a plant survey was conducted in the beneficiation, smelting, and tailings areas of the realgar mining area. The dominant plants were identified, and rhizosphere soil samples were collected. The fungal diversity in the rhizosphere soil was investigated using 18S rDNA high-throughput sequencing technology. Further, (1) the dominant phyla, classes, and orders of rhizosphere soil fungi in different functional zones; (2) the similarities and differences in the composition and distribution of fungal communities in rhizosphere soil across different functional zones; and (3) the relationship between fungal diversity in the rhizosphere soil and soil environmental factors were determined. The findings of this study are anticipated to enhance the understanding of the fungal community diversity in As-contaminated areas, particular focusing on fungi in symbiosis with plants, and establish a theoretical foundation for the future advancement of plant–fungal combined remediation technologies in As contaminated areas.

## 2 Materials and methods

### 2.1 Overview of the study area

The study area is located in a highly As-contaminated area, the realgar mining area, northwest of Shimen County, Changde City, Hunan Province (**Supplementary Figure S1**). It has a subtropical monsoon climate, with an average annual precipitation of 1567.3 mm and an average annual temperature of 18.4°C.

### 2.2 Investigation and analysis of plants and fungi in the study area

Based on detailed investigation of realgar mining area and literature on heavy arsenic contamination (Zeng et al., 2016), samples were collected from three areas: Shihuangsi village (W-B) nearby beneficiation area, Heshan village (W-S) nearby smelting area, and Linkuang village (W-T) nearby tailings area (**Supplementary Table S1**). In each area, we randomly set up 10 shrub quadrats, each measuring 10 m × 10 m. Within each of these quadrats, we further set up 1 small quadrats, each measuring 1 m × 1 m, for the investigation of herbaceous plants. These plots were set up in locations with similar slope positions and aspects with dimensions. Subsequently, we carried out a survey of the plant distribution to determine the dominant native plants in As-contaminated areas. The proportion of a certain plant in the study area is shown in Equation (1).


(1) $$\begin{array}{c} Proportion\;of\;a\;certain\;plant\;in\;the\;study\;area\;\left(\%\right)\\ =\frac{Pi}{\sum_{1}^{n}Si}\;\times 100\% \end{array}$$

Where *Pi* (m^2^) is the distribution area of a certain plant in the study area, *Si* (m^2^) is the area of each quadrat, *n* is the numbers of sampling point.

After the dominant native plants were identified, the rhizospheric soil of the dominant plants was randomly collected at 3 points in the W-B, W-S and W-T areas. A total of 2 kg of rhizosphere soil (0–20 cm) of the dominant plants was collected, mixed, and divided into two portions. One portion was stored in a 4°C mini fridge (SAST, China) and transported to the laboratory for the analysis of physicochemical properties. The other portion of soil sample was placed in a liquid nitrogen tank and immediately transported to the laboratory for microbial diversity analysis.

### 2.3 Soil property analysis

The soil samples were air-dried, ground, and sieved through a 2 mm sieve, and soil property analysis was conducted. In a beaker containing 10 g of soil, 25 mL of deionised water was added, magnetically stirred for 10 min, and allowed to stand for 30 min. The pH of the supernatant was measured using a pH meter (PHBJ-260, Leici, China). The air-dried soil was sieved through a 100-mesh sieve and divided into two portions. One portion was treated with hydrochloric acid (HCl) to eliminate inorganic carbon, followed by air-drying, grinding, and analysis for total organic carbon (TOC) using an elemental analyser (Elementar variomacro EL, Germany), and the other portion was analysed for nitrogen (N) and sulphur (S) contents by elemental analyser. Available potassium (AK) was quantified using flame photometry (AA-6880, Shimadzu, Shanghai, China). Soil-available phosphorus (AP) was determined using the sodium bicarbonate (NaHCO_3_) extraction method (Duboc et al., 2022).

The measurement of total arsenic (TAs) and total antimony (TSb) in soil samples was performed as follows: A solution of 8 mL acid mixture (nitric acid, hydrochloric acid, and hydrofluoric acid in a 5:2:1 ratio) was added to a PTFE tube containing 50 mg of soil and allowed to stand overnight, followed by digestion for 2 h using a microwave system (CEM, MARs5, USA). After cooling the digestion tube to 20°C, the sample was transferred to a 90°C water bath until the solution became clear. The solution from the tube was then transferred to a 50 mL centrifuge tube and filtered through a 0.45 μm polyethersulfone membrane. The volume was adjusted to 50 mL by adding 5% hydrochloric acid. The concentrations of As and Sb were measured using an inductively coupled plasma optical emission spectrometer (ICP-OES, Agilent 5900, USA). Quality control was maintained throughout the analysis as follows. The recovery rate of As in soil was 70.7%–110.0%, and that of Sb was 80.0%–120.0%. Standard solutions of As and Sb were calibrated by standard curves, and the correlation coefficient (r) was greater than 0.995. The relative standard deviation (rsd) of repeated samples in soil was less than 10%.

### 2.4 Determination of soil microbial diversity using 18S rDNA technology

Total DNA extraction for different microbial colonies was performed using DNA extraction kits (TianGen Biotech, Beijing, China). The primers for fungal 18S rDNA gene amplification were 817F: 5′-GCCTCCCTCGCGCCATCAG (10 bp MID) CAGCCGCGGTAATTCCAGCT-3′ and 1196R: 5′-GCCTTGCCAGCCCGCTCAG GTTTCCCGTAAGGCGCCGAA-3′. Samples were amplified using PCR (ABI GenAmp^®^9700, USA) with barcode-specific primers and analysed by million agarose gel electrophoresis (E-Gel Power Snap, Thermo Fisher Scientific, America). Quantitative detection of PCR products was performed using a fluorescent blue quantitative system (QuantiFlourTM-ST, USA), and the products were mixed in different proportions according to sequencing needs. The PCR products were amplified to construct a MiSeq library template, and single-stranded DNA was obtained via alkaline denaturation. DNA fragments complementary to the primers were fixed onto the chip, and complementary DNA fragments from other primers were randomly generated, creating relevant bridges. Amplification bridges were formed to produce DNA clusters. Linear biased DNA amplicons became single-stranded. Four types of fluorescent-labelled dNTPs and modified DNA polymerase were added to the PCR, synthesising a single base in each loop. The nucleotide sequence of each template was read from the template sequence of each reaction. The “terminator” and “fluorophore” were chemically cleavaged to restore 3′ terminal activity, enabling the synthesis of the next nucleotide. Based on the fluorescence signal result, the DNA template sequence was obtained and identified. The data presented in the study are deposited in the NCBI repository, accession number PRJNA1334568.

### 2.5 Statistical analysis

Data analysis was performed using the SPSS^®^19.0 software package. Analysis of variance (ANOVA) was used to assess the differences between the mean values of soil characteristics, and the t-test was applied for comparison at the 0.05 probability level (*p*=0.05). Observed richness indices such as Chao, Shannon, and Good’s coverage were calculated using Mothur (version 1.80.2). Non-metric multidimensional scaling (NMDS) and redundancy (RDA) analyses were performed using Canoco 5 software. The figures were processed using Origin^®^2019b.

## 3 Results and discussion

### 3.1 Identification of dominant native plant in arsenic mining area

Plant community composition is shown in **Table 1**, there are 23 species in As-contaminated areas, belonging to 13 families and 23 generas. Among them, there are 4 species of trees, 1 species of shrubs and 18 species of herbs. In herbaceous plants, characteristics of the most diverse and widely distributed plant species in Poaceae. Among them, the distribution area of Poaceae accounted for 16.2% of the total quadrat area. Based on the comprehensive analysis of plant community composition and distribution, Poaceae are identified as the dominant native plant category in As-contaminated soil. Further to analysis distribution of Poaceae revealed that the distribution area of *Imperata cylindrica* (L.) Beauv in As-contaminated soil accounts for 5.5% of the total sample quadrat area. Concurrently, extensive researches indicate that *Imperata cylindrica* (L.) Beauv also serves as a primary pioneer plant in other high-concentration abandoned heavy metal mining area. It can widely exist in mining areas and simultaneously accumulate large amounts of heavy metals such as chromium and copper (Fu et al., 2016; Zheng et al., 2019; Mao et al., 2024). Considering the significant proportion of *Imperata cylindrica* (L.) Beauv among herbaceous plants, it can be considered a potentially dominant native species for ecological remediation in As-contaminated soil. In addition, *Pteris vittata* (L.) accounts for 7.2% of the total sample quadrat area. The Pteridaceae to which *Pteris vittata* (L.) belongs, is not dominant native family in As-contaminated soil, a large number of articles have demonstrated that *Pteris vittata* (L.) is a hyperaccumulator of As (Nguyen et al., 2011; Bai et al., 2023). Therefore, *Pteris vittata* (L.) is regarded as an excellent candidate for phytoremediation of highly arsenic-contaminated soils. In summary, considering the distribution of plants in the community and special ecological functions, the potential dominant native species for ecological remediation in high soil As concentration areas are *Pteris vittata* (L.) and *Imperata cylindrica* (L.) Beauv.

**Table 1:** Components and proportion of plant community in As-contaminated areas.

Ecotype	Family	Genus	Species	Proportion (%)
Tree layer	Pinaceae	*Pinus*	*Pinus massoniana* Lamb.	4.2
	Moraceae	*Broussonetia*	*Broussonetia papyrifera*	3.7
	Lauraceae	*Cinnamomum*	*Cinnamomum camphora* (Linn) Presl.	3.1
	Sapindaceae	*Koelreuteria*	*Koelreuteria paniculata*	2.8
Shrub layer	Urticaceae	*Urticaceae*	*Boehmeria nivea* (L.) Gaudich.	2.3
Herb layer	Pteridaceae	*Pteris*	*Pteris vittata* (L.)	7.2
	Poaceae	*Imperata*	*Imperata cylindrica* (L.) Beauv.	5.5
	Poaceae	*Pennisetum*	*Pennisetum alopecuroides*	3.7
	Poaceae	*Cynodon*	*Cynodon dactylon* (L.) Pers.	1.3
	Poaceae	*Eleusine*	*Eleusine indica*	2.2
	Poaceae	*Poaceae*	*Setaria viridis* (L.) Beauv.	1.9
	Poaceae	*Digitaria*	*Digitaria sanguinalis* (L.) Scop.	0.9
	Poaceae	*Arthraxon*	*Arthraxon hispidus* (Trin.) Makino	0.7
	Asteraceae	*Conyza*	*Conyza canadensis* (L.) Crong.	0.4
	Asteraceae	*Bidens*	*Bidens pilosa* L.	0.3
	Fabaceae	*Glycine max*	*Glycine soja* Sieb. el Zucc.	0.6
	Fabaceae	*Trifolium*	*Trifolium repens*	0.2
	Plantaginaceae	*Plantago*	*Plantago depressa* Willd.	0.2
	Cyperaceae	*Carex*	*Carex* spp.	0.1
	Phytolaccaceae	*Phytolacca*	*Phytolacca acinos* Roxb	0.1
	Polygonaceae	*Rumex*	*Rumex acetosa* L.	0.7
	Rosaceae	*Rubus Linn*	*Rubus corchorifolius* L. f.	0.8
	Euphorbiaceae	*Acalypha*	*Acalypha australis* L.	1.0

### 3.2 Characteristics of arsenic and associated metal content in different regions

**Figure 1:**
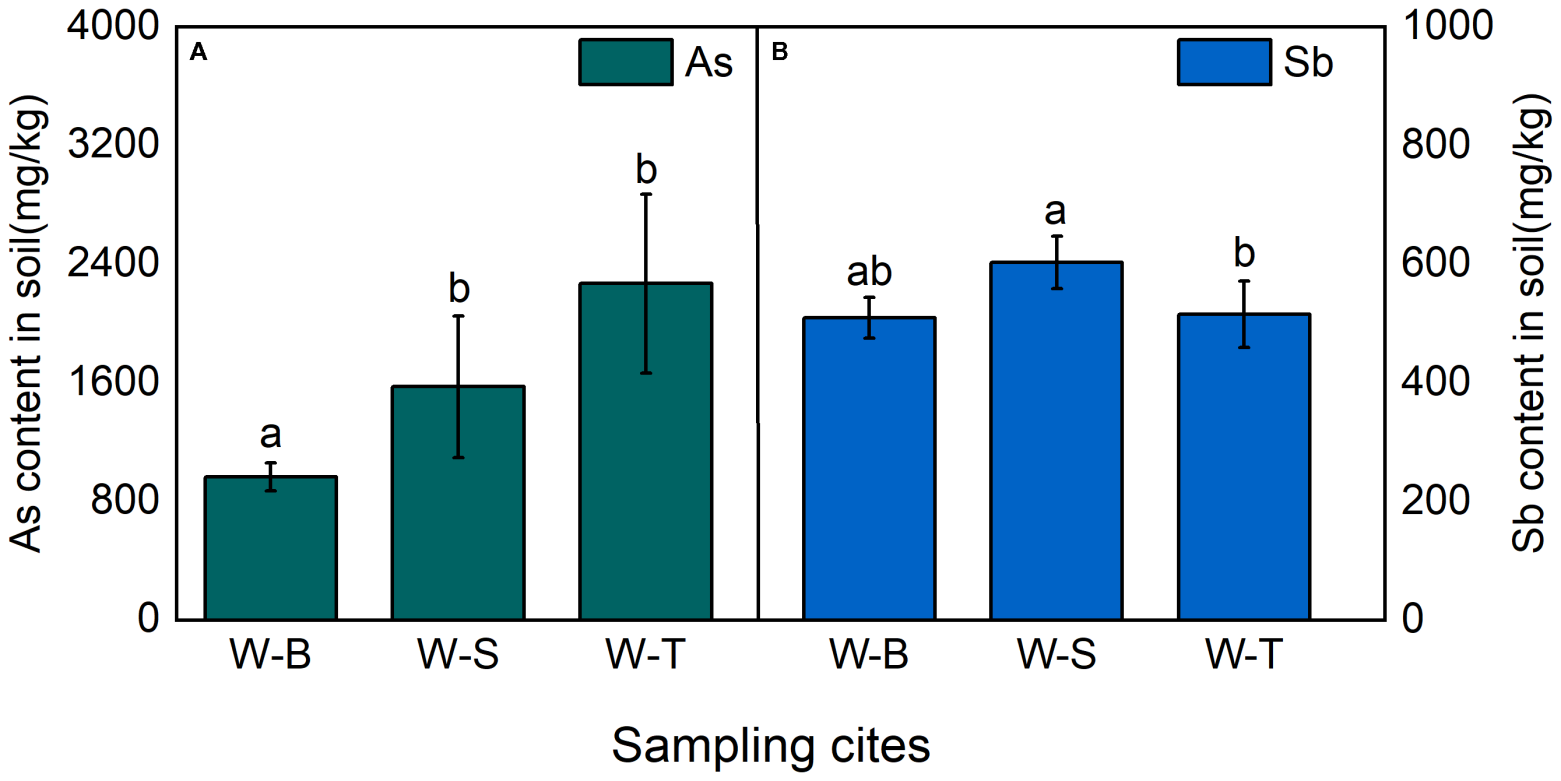
As and Sb contents of soil in As-contaminated areas, **(A)** As content in soil; **(B)** Sb content in soil. Shihuangsi village (W-B); Heshan village (W-S); Linkuang village (W-T).

As in soil can negatively impact plant physiology and growth, thereby reducing the efficiency of phytoremediation for As-contaminated soil. Therefore, we measure the concentrations of As in rhizospheric soil of *Pteris vittata* (L.) and *Imperata cylindrica* (L.) Beauv. The heavy metal content in soils from different sample points of As-contaminated areas is shown in **Figure 1A**. The average concentrations of As in the soils of W-B, W-S, and W-T areas were 964.69, 1574.04, and 2268.30 mg/kg, respectively, which far exceed the TAs concentration limit set by the European union soil guidelines (10 mg/kg) (Anawar et al., 2006). This indicated that soils in all sample points were severely contaminated with As. There were significant differences in the soil As content across different sample points, with W-S area exhibiting the highest average As concentration, followed by W-S area. Both areas were1.63 and 2.35 times higher than the As concentration (*p* < 0.05) in W-B area, respectively. As and Sb usually exist together (Mi et al., 2023). Hence, we further investigated the Sb content in soils from the different sample points. The results (**Figure 1B**) indicated that the soil Sb concentrations from W-B, W-S, and W-T areas were 509.73, 602.56, and 515.62 mg/kg, respectively. These concentrations exceeded the soil safety limit established by the WHO (36 mg/kg) by 14.16, 16.74, and 14.32 times, respectively (Jin and Peng, 2024). Sb concentrations in soil of W-S and W-T areas were higher than in W-B area (*p* < 0.05). The above results showed that the heavy metal content in soils of Heshan village and Linkuang village was significantly higher than that in W-B Shihuangsi village. This could be attributed to the fact that W-B area near by the beneficiation area involves only physical processing of the ore, resulting in a reduction in ore volume. Consequently, a large number of heavy metals still exist in mineral forms, such as AsS, As_2_S_3_, or FeAsS, without being released into the soil (Kim et al., 2014; Liu et al., 2022). In contrast, W-S near by the smelting and W-T nearby tailings areas, respectively. The ore in the W-S and W-T area undergo further physical or chemical processing, which facilitates the release of heavy metals and their accumulation in the soils (Timofeev et al., 2018).

### 3.3 Sequencing data and fungal taxonomic richness

The forms and content of As and associated metal in the soil can significantly affect the composition and structure of the dominant plant rhizosphere fungal communities (Chodak et al., 2013; Tang et al., 2022). Lin et al. (2020) analysed the primary factors influencing soil microbial diversity by collecting soil samples from 23 regions, revealing a significant negative correlation between fungal community abundance and evenness in soil and cadmium (Cd) content. We performed operational taxonomic unit (OTU) classification for all sequences at a 97% similarity level, and the results indicated that the average number of OTUs in W-B, W-S, and W-T area were 3401, 2758, and 3571, respectively (**Supplementary Figure S2**). Therefore, we hypothesised that the fungal diversity in W-B and W-T areas may be higher than in W-S area.

**Figure 2:**
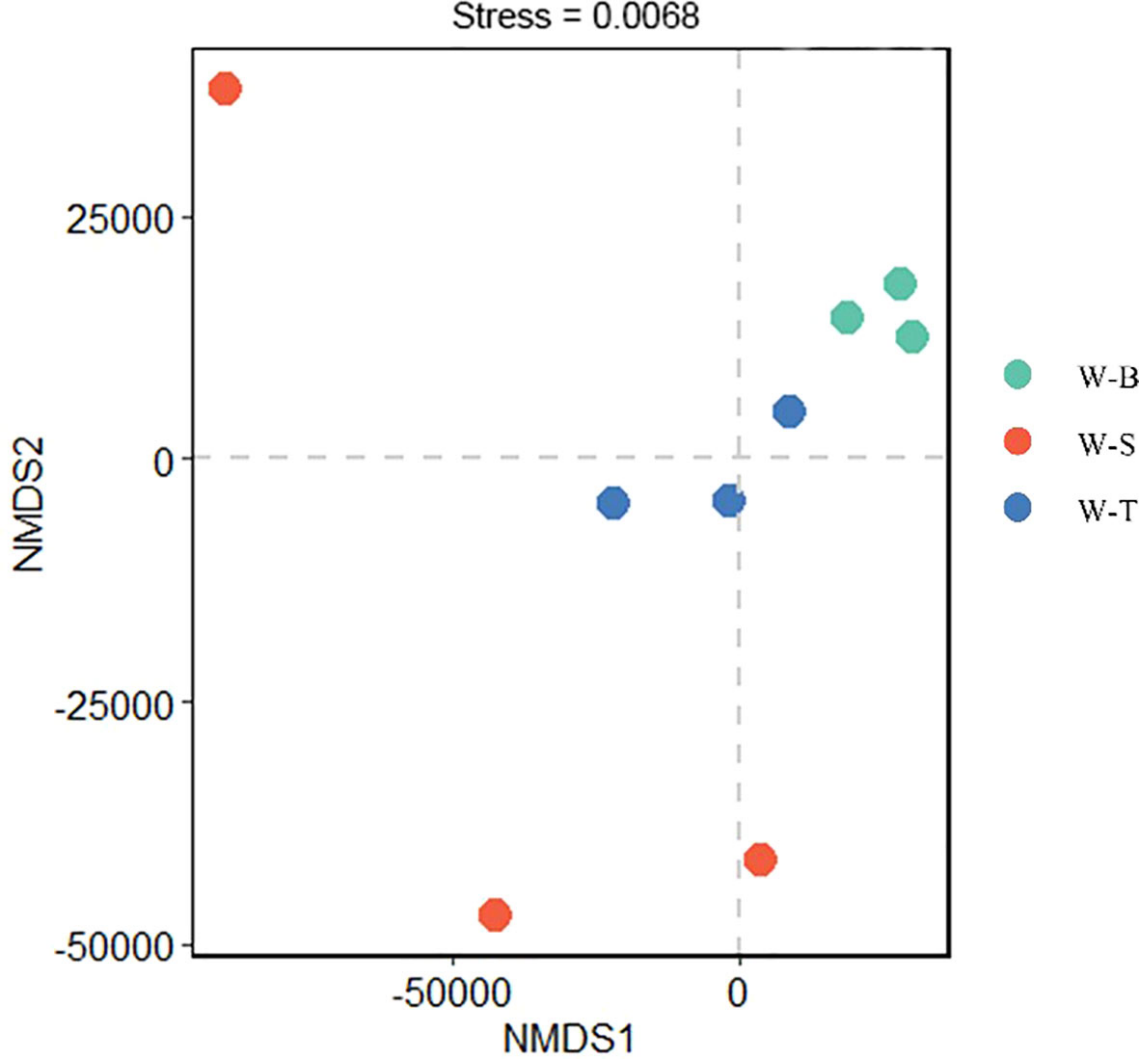
NMDS plot. Shihuangsi village (W-B); Heshan village (W-S); Linkuang village (W-T).

To further validate this hypothesis, we examined the α-diversity of fungi in the different sample points. The sample coverage results are shown in **Table 2**, indicating no significant differences in coverage between the three sample points. The coverage rate reached 90%, indicating that the sequencing results effectively portray the fungal community diversity characteristics of the three sample points. We further analysed the Chao index to estimate the number of OTUs in the samples, which can evaluate the total species number. The results (**Table 2**) indicated that the Chao index decreased in the following order: W-T area > W-B area > W-S area, with the Chao index for W-S area being only 80% of that in W-B and W-T areas. This suggested that the total number of fungal species W-S area is lower than in the other two sample points; however, there was no significant difference in the total fungal species between the three sample points (*p* < 0.05). Chao index in the uncontaminated area reported by Xin (2025) exceeded 10000, significantly higher than the Chao indices at the three sampling sites in our study, which indicates diversity index of fungi in uncontaminated area is significant greater than that observed at the three sampling sites. We also analysed the Shannon index to evaluate the relative abundance (evenness) of microbes in the samples. The results (**Table 2**) showed that the relative abundance of fungi is highest in W-T area, followed by W-B area, with W-S area having the lowest fungal relative abundance. The Shannon index of W-T area was significantly higher than that of W-S area (*p* < 0.05); however, there was no significant difference in fungal relative abundance between W-T and W-B areas. Similarly, the Shannon index for fungi in the uncontaminated area exceeded 7.5, surpassing the corresponding indices at the three sampling sites. This indicates a higher relative abundance (evenness) of fungal species in the uncontaminated sites. This may be attributable to the selective pressure exerted by high heavy metal concentrations on fungal communities, leading to a more concentrated distribution of species within communities exhibiting greater tolerance to heavy metals, thereby reducing relative abundance (evenness). In summary, the α-diversity was highest in W-T area, followed by W-B area, and lowest in W-S area, which was consistent with the OTU classification results of the three sample points (**Supplementary Figure S2**). In addition to the α-diversity of fungal communities, we used Non-metric multidimensional scaling (NMDS) based on Bray–Curtis distances to determine the β-diversity of fungal communities in different sample points. NMDS is an ordination technique used to analyse species composition data and is often used for interpreting the microbial community distributions (Li et al., 2022; Sajjad et al., 2024). The NMDS results are shown in **Figure 2**, with a stress value of 0.0068 (<0.05), indicating that the results are highly representative. W-B area was in close proximity to W-T area and farther from W-S area, indicating that the fungal diversity and composition of W-B area closely resemble those of W-T area.

**Table 2:** Diversity of fungi in different sample points.

Sample point	Chao	Shannon	Coverage
W-B	7020±2834a	7.12±0.43ab	0.90±0.05a
W-S	5670±2227a	6.08±0.69a	0.95±0.06a
W-T	7070±1744a	7.29±0.17b	0.93±0.05a

Shihuangsi village (W-B); Heshan village (W-S); Linkuang village (W-T).

### 3.4 Fungal community composition and distribution in the different sample points

**Figure 3:**
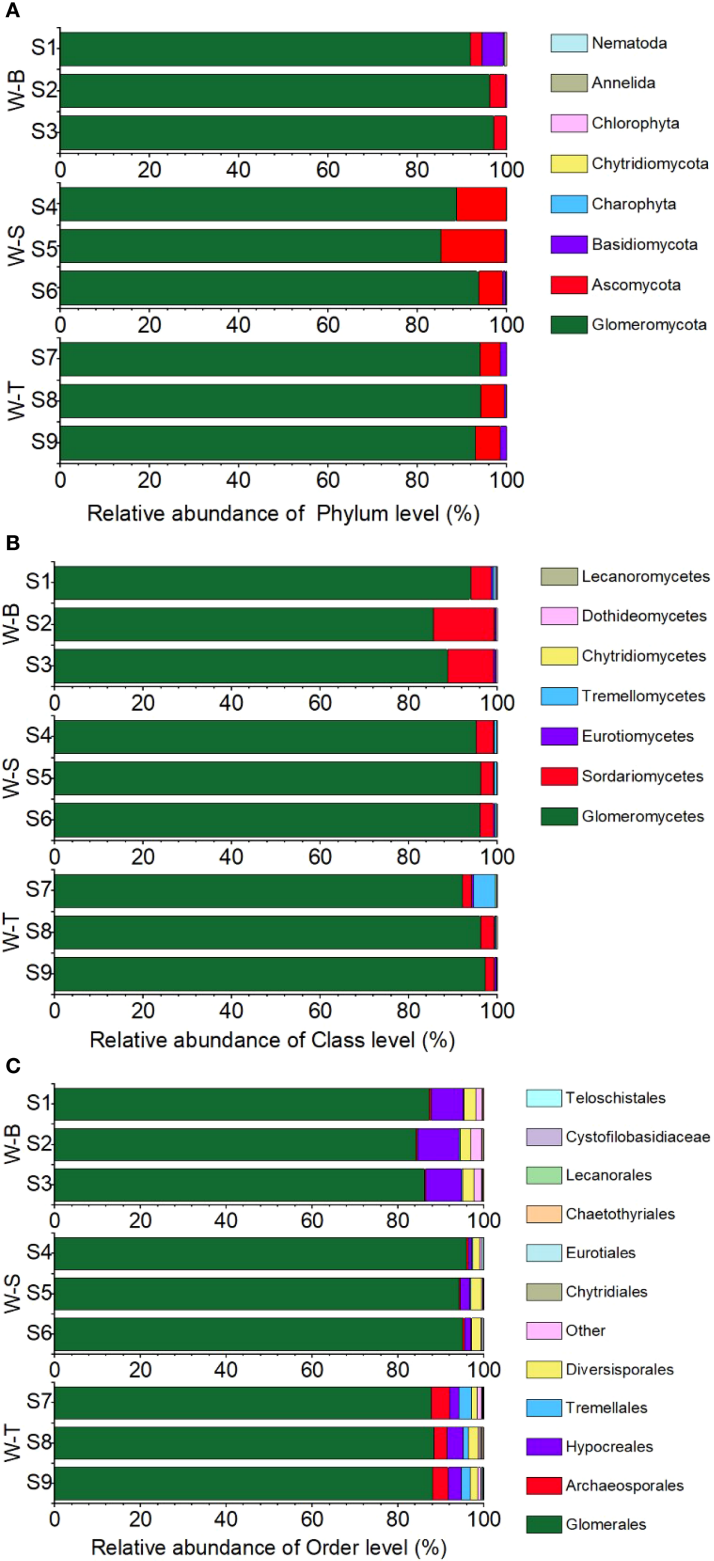
Fungi composition and abundance for each pollution level at phylum **(A)**, class **(B)** and order levels **(C)**. Shihuangsi village (W-B); Heshan village (W-S); Linkuang village (W-T).

To further show the microbial composition in the different sample points, we analysed the fungal composition at the phylum, class, and order levels in the soil samples from the three sample points. As shown in **Figure 3A**, eight common fungal phyla were detected in the experimental soils of the three sample points. Among them, the phyla *Glomeromycota*, *Ascomycota*, and *Basidiomycota* exhibited higher relative abundances in W-B, W-S, and W-T areas. This indicates that these three phyla comprise the dominant microorganisms in As-contaminated areas, with the relative abundance of *Glomeromycota* exceeding 80% in all three sample points. *Glomeromycota* constitutes the primary microbial phylum to which AMF belong. It suggests that AMF communities may dominate fungal community in soil. At the phylum level, the relative abundance of non-AMF-associated *Ascomycota* and *Basidiomycota* was highest in fungal communities from uncontaminated site, followed by the *Glomeromycota* (10%). Notably, the relative abundances of *Ascomycota* and *Basidiomycota* were significantly higher than that of *Glomeromycota*, which may be due to the selective pressure exerted by heavy metals in contaminated areas upon microbial communities. In the high concentration of arsenic, *Glomeromycota* exhibits greater adaptability compared to the phyla *Ascomycota* and *Basidiomycota*. Furthermore, variations in fungal community diversity were observed among the different sample points. For example, the relative abundance of *Glomeromycota* in the W-S and W-T areas were lower than in W-B area, possibly due to the stronger inhibitory effect of high concentrations of As on the growth of specific *Glomeromycota* fungi (Yang et al., 2023).

At the class level, *Glomeromycetes* belonging to the phylum *Glomeromycota* exhibited the highest relative abundance at the class level in all three sample points, with relative abundances of 96.24%, 89.41%, and 94.81% in W-B, W-S, and W-T areas, respectively (**Figure 3B**). *Sordariomycetes* exhibited the second highest relative abundance in all three sample points. *Glomeromycetes* and *Sordariomycetes* accounted for 95% of the microbial classes in the three sample points, suggesting that, at the class level, *Glomeromycetes* and *Sordariomycetes* are the dominant microorganisms in As-contaminated areas. Similarly, variations were observed in the fungal diversity at the class level between the different sample points. The relative abundance of *Glomeromycetes* in the W-S and W-T areas were higher than in W-B area. Interestingly, the class *Glomeromycetes*, which had the highest relative abundance at the class level, belongs to *Glomeromycota* and is also a class of arbuscular mycorrhizal fungi (AMF). AMF exhibit wide distribution and forms symbiotic relationships with plants. It substantially improves plant survival under stress by enhancing the plant’s nutrient absorption, improves soil structure, and regulates the expression of proteins related to plant stress resistance (Allsup et al., 2023; Martin and van der Heijden, 2024). Even in environments with severe heavy metal contamination, wastewater irrigation, and salt marshes, AMF exhibit widespread distribution and plays an essential ecological role (Arriagada et al., 2009; Sonjak et al., 2009; Marro et al., 2022). Furthermore, *Sordariomycetes*, belonging to the phylum *Ascomycota*, plays a crucial role in the decomposition of organic matter and in soil nutrient cycling (Zhang et al., 2006). These results indicate that functional microorganisms that play a key role in As-contaminated areas exhibit a high level of tolerance to heavy metal pollution.

At the order level, a total of 32 orders were identified in the soil samples, among which *Glomerales*, *Archaeosporales*, *Hypocreales*, and *Diversisporales* were common to all three sample points (**Figure 3C**). In the W-B and W-T areas, *Glomerales*, *Hypocreales*, and *Diversisporales* were most abundant, with relative abundances of 98.66% and 96.99%, respectively. In the W-S area, the three orders with the highest relative abundance were *Glomerales*, *Archaeosporales*, and *Hypocreales*. *Glomerales* was the most abundant order in all three sample points and is an important order in the AMF classification, further supporting the idea that AMF shows a high level of tolerance to As contamination. Additionally, the study indicated a marked decrease in the relative abundance of *Glomerales* in the W-S and W-T areas compared to W-B area (**Figure 3C**). This divergence could be attributed to the increased susceptibility of *Glomerales* to elevated levels of heavy metal contamination (Dai et al., 2012).

### 3.5 Influence of soil physicochemical properties on microbial diversity

**Figure 4:**
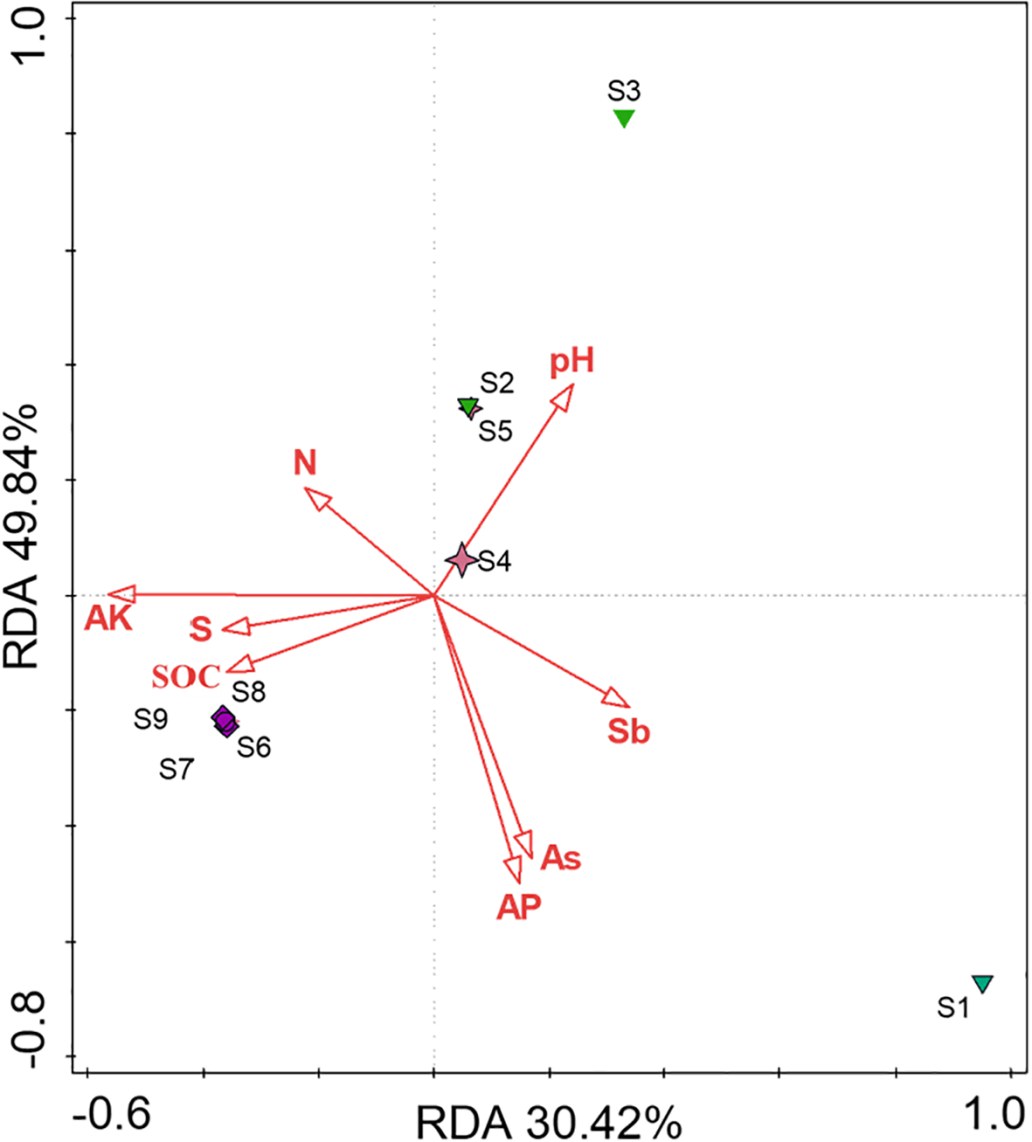
RDA of the relationship between fungi species and soil physicochemical properties.

To further explore the main environmental factors influencing fungal diversity in the different sample points, this study used redundancy analysis (RDA) to validate the correlation between eight environmental factors and soil fungal diversity. As a constrained linear by gradient sorting method, RDA can reflect the influence of environmental factors on species composition and also express the explanatory value of each environmental factor. The degree of correlation between the environmental factor arrows and microbial arrows is represented by the cosine of the angle between them. The results of the RDA are shown in **Figure 4**. Overall, the soil AP, AK, and As contents had the most notable effects on fungal diversity in each functional area. Additionally, in the W-S and W-T areas, soil pH, AP, and As had the most notable effects on fungal diversity. In W-B area, SOC had the greatest impact on fungal diversity. These results indicate variations in the environmental factors influencing microbial diversity across the different sample points. In both W-S and W-T areas, fungal diversity was positively correlated with soil pH and negatively correlated with the concentrations of AP and As. This relationship may be associated with the dominance of AMF in the fungal communities of the W-S and W-T areas. AMF is an oligotrophic symbiotic microorganism that provides nutrients for plant growth by absorbing more phosphorus from the soil and exchanging it for plant carbon sources (Duan et al., 2024). At elevated soil AP concentrations, the phosphorus uptake by plants through their roots is sufficient to fulfil their growth requirements. This results in reduced carbon supply from plants to AMF, thereby leading to a decrease in the abundance and diversity of AMF (Olsson et al., 2010; Basyal and Emery, 2021). Furthermore, heavy metals are another important factor influencing AMF diversity. Excessive concentrations of heavy metals can have toxic effects on AMF, thereby affecting the composition and structure of AMF communities (Betancur-Agudel et al., Chen et al., 2023). This could potentially elucidate the negative relationships among AP, As, and AMF. Soil pH influences the form of As and phosphorus, indirectly affecting the composition and structure of the AMF community. When soil pH decreases, insoluble phosphates are released, increasing the AP content. Meanwhile, lowered pH can lead to the release of As bound to carbonates in the soil, thereby increasing the concentration of bioavailable As and increasing the toxic effects of heavy metals on AMF, ultimately reducing its diversity. Therefore, there is a positive correlation between soil pH and microbial community structure. In W-B area, soil SOC had the most notable effect on fungal diversity, which is consistent with the findings of Bi et al. (2020). This is likely because the germination of AMF spores and hyphae necessitates elevated levels of SOC. The soil in W-B area is primarily composed of As_2_S_3_ minerals and has an extremely low organic carbon content. This deficiency in SOC inhibits the growth of spores and hyphae, rendering SOC the primary factor influencing the composition of the AMF community in the rhizosphere soil or roots (Stürmer et al., 2018; Luo et al., 2019).

## 4 Conclusions

This study investigated the distribution of plant community composition in highly As contaminated area, the realgar mining area, in Shimen county, Hunan province. The results showed that *Pteris vittata* (L.) and *Imperata cylindrica* (L.) Beauv. are the dominant native species in this area. In addition, this study investigated the diversity of plant-symbiotic fungi in this area, among which the relative abundances of three fungal phyla, *Glomeromycota*, *Ascomycota*, and *Basidiomycota*, were highest in the W-S, W-T, and W-B areas. *Glomerales* (the order to which AMF belongs) was identified as the dominant order in highly As-contaminated areas. Furthermore, As and AP were negatively correlated in the W-S and W-T areas and showed a significant positive correlation with soil pH. This could indicate that bioavailable As contamination disrupted the ecological composition of the fungi. In W-B area, organic carbon had the most notable effect on fungal diversity, which could be attributed to the low organic carbon content in W-B area, and is insufficient to meet the carbon source requirements for the growth of *Glomerales*. These findings offer valuable insights for the development of future plant–fungal combined ecological remediation technologies for As-contaminated soil, especially the soil with high As concentration. This study provides a potential strategy to utilize plant-symbiotic fungi (AMF) to reduce As toxicity to plants. Furthermore, some studies indicate that during practical soil remediation for heavy metals, the efficacy of AMF is influenced by factors such as ambient temperature, soil particle size, and other composite pollutants (e.g., plastics, chemical fertilisers, and pesticides). Previous studies have reported that combined pollution from heavy metals and microplastics can affect the diversity of AMF communities. Based on the above contents, when applying AMF to practical arsenic (As)-contaminated soil remediation in the future, it will be necessary to comprehensively consider actual environmental conditions and scientifically and rationally utilise locally dominant AMF. This approach will effectively enhance the application efficacy of AMF in soil remediation.

## Data Availability

The data presented in the study are deposited in the NCBI repository, accession number PRJNA1334568.
