# Supplementary figures and images for: Revealing the critical role of in-situ plant and microbe community structure in remediation of typical high-arsenic soil through molecular analysis
Source: Front Plant Sci. 2025 Oct 10;16:1608933. doi: 10.3389/fpls.2025.1608933 (PMC12550955; doi:10.3389/fpls.2025.1608933)

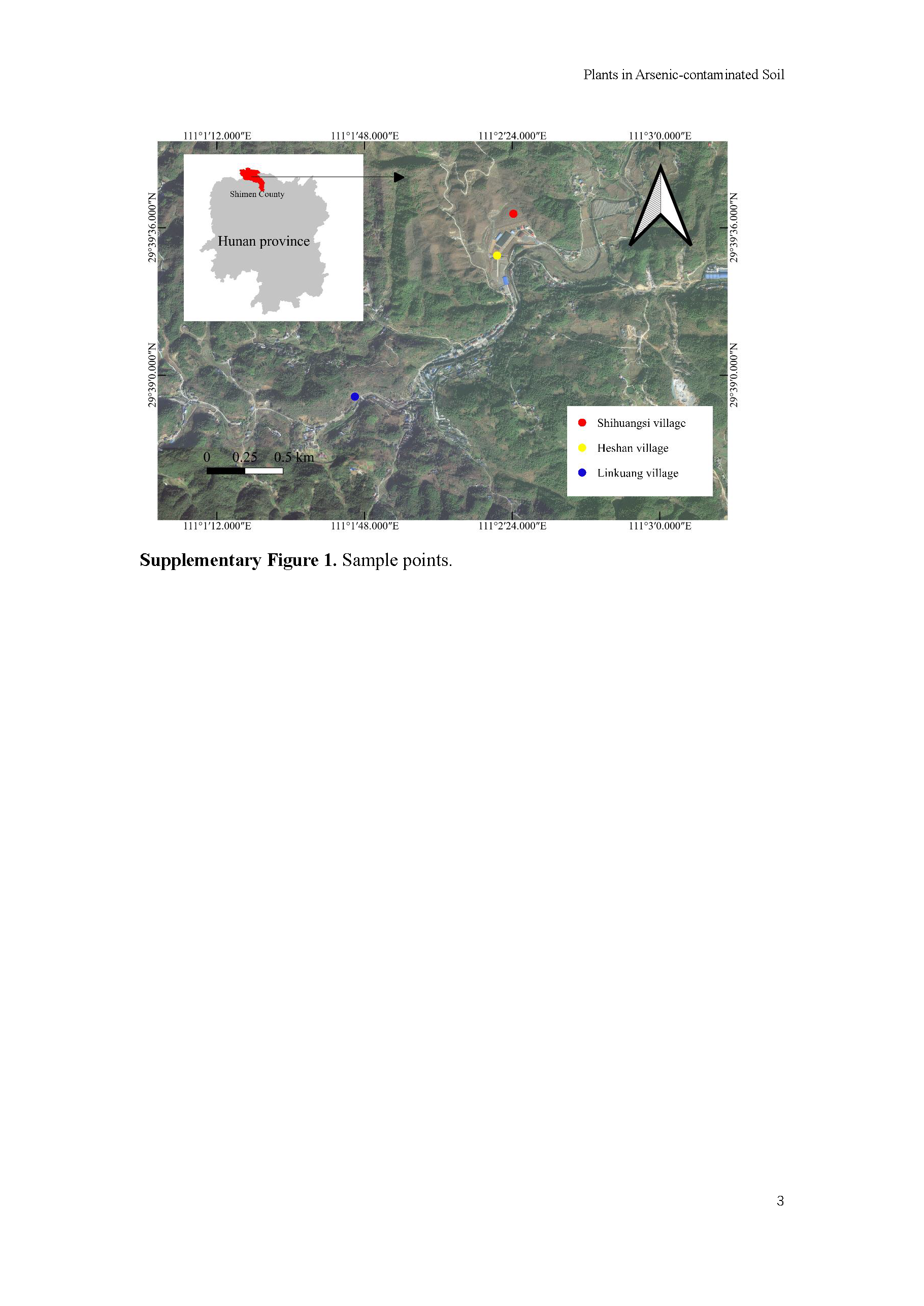

Supplement: Supplementary file 1 [file Image1.tif]

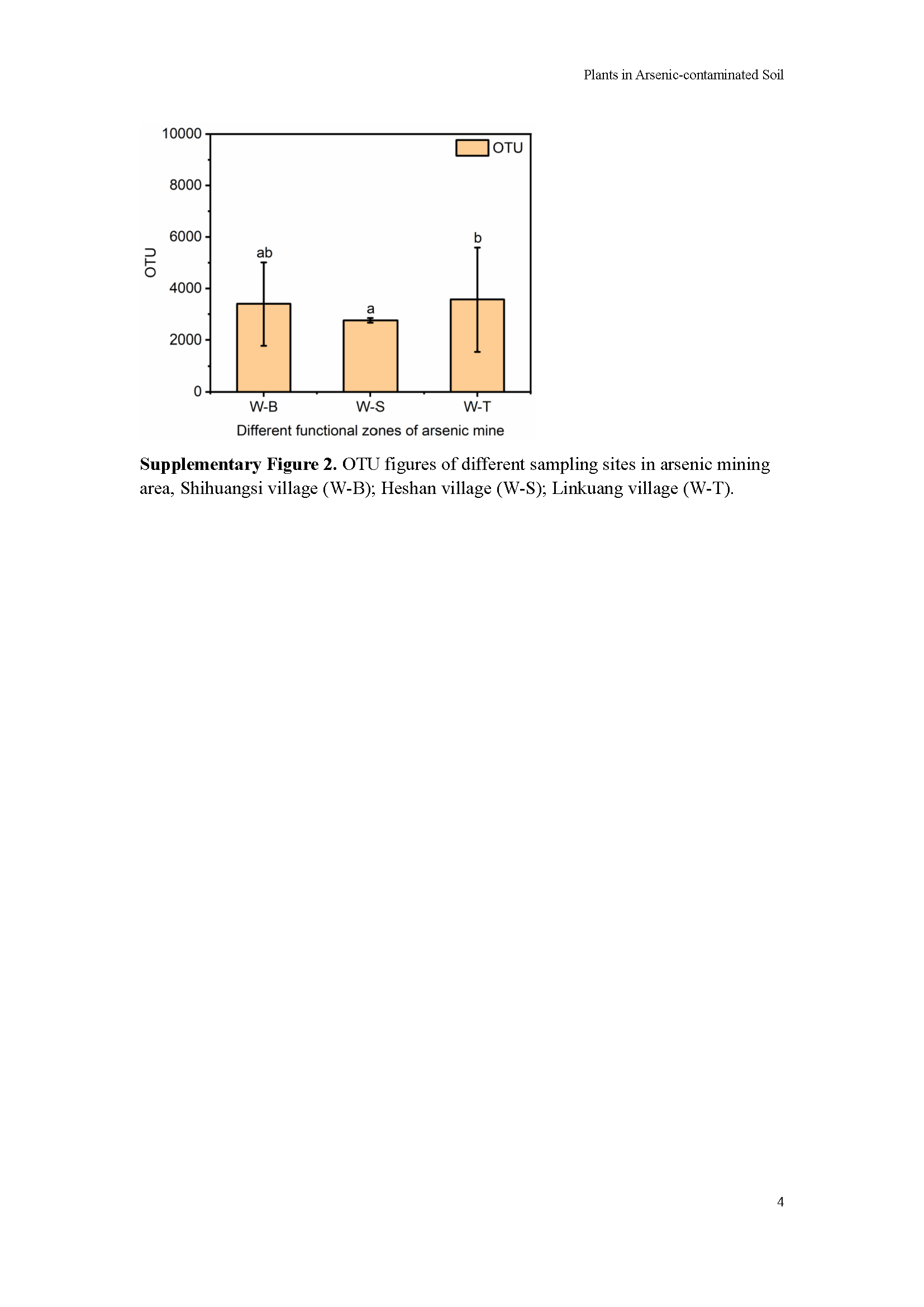

Supplement: Supplementary file 2 [file Image2.tif]
