# Supplementary material for: Revealing the critical role of in-situ plant and microbe community structure in remediation of typical high-arsenic soil through molecular analysis
Source: Front Plant Sci. 2025 Oct 10;16:1608933. doi: 10.3389/fpls.2025.1608933 (PMC12550955; doi:10.3389/fpls.2025.1608933)
Supplement: Supplementary file 3 [file Table1.docx]

**Supplementary Table 1.** Longitude and latitude of sampling sites.

| Sampling sites | Latitude (N) | Longitude (E) |
| --- | --- | --- |
| W-B | 29°39'28.8639" | 111°02'45.1177" |
| W-S | 29°39'18.3763" | 111°02'39.5735" |
| W-T | 29°38'44.3430" | 111°02'04.3660" |

Shihuangsi village (W-B); Heshan village (W-S); Linkuang village (W-T).
